# Degree of conversion of experimental resin composites containing bioactive glass 45S5: the effect of post-cure heating

**DOI:** 10.1038/s41598-019-54035-y

**Published:** 2019-11-21

**Authors:** Matej Par, Nika Spanovic, Tobias T. Tauböck, Thomas Attin, Zrinka Tarle

**Affiliations:** 1 Department of Conservative and Preventive Dentistry, Center for Dental Medicine, University of Zurich, Plattenstrasse 11, Zurich, Switzerland; 2 Community Health Center, Hirceva 1, Zagreb, Croatia; 3 Department of Endodontics and Restorative Dentistry, School of Dental Medicine, University of Zagreb, Gunduliceva 5, Zagreb, Croatia

**Keywords:** Biomedical materials, Bioinspired materials

## Abstract

Resin composites containing reinforcing inert glass fillers combined with bioactive glass (BG) can aid in the prevention of secondary caries, which is a major cause of failure of contemporary composite restorations. A series of previous studies on experimental resin composites filled with BG 45S5 has demonstrated that methacrylate resin polymerization can be impaired by the addition of unsilanized BG, leading to lower degrees of conversion (DC). In order to distinguish whether the polymerization inhibition is caused by a direct (temperature-independent) effect of BG or an indirect (temperature-dependent) effect of restricted mobility of reactive species, this study used Raman spectroscopy to evaluate the DC values of experimental composites post-cured at 37 °C and 150 °C. The potential of BG to adversely affect DC was highly dependent on the resin system. The highest DC reduction was observed in the resin system based on ethoxylated bisphenol A dimethacrylate (Bis-EMA), followed by bisphenol A glycidyl methacrylate (Bis-GMA). In contrast, the DC for urethane dimethacrylate (UDMA) was not compromised by BG. Increasing the mobility of reactive species by heating at 150 °C showed limited potential for increasing the DC in the Bis-EMA and Bis-GMA resin systems, indicating a direct inhibitory effect of BG on polymerization.

## Introduction

Bioactive resin composites are an interesting alternative to inert composites, which currently dominate dental practice^1^. The research on bioactive composites is driven by the need to prevent secondary caries, which is one of the major causes of composite restoration failure^2^. The usual approach for rendering a composite material bioactive is to partially replace its inert glass fillers with remineralizing fillers, for example, bioactive glass (BG)^3,4^. Experimental resin composites produced by admixing various types of BG into methacrylate resins have demonstrated multiple beneficial features, including hydroxyapatite precipitation^3^, sealing of marginal gaps^5^, local antibacterial effects^6^, prevention of dental hard tissue demineralization^7^, induction of remineralization^8^, and protection of the hybrid layer through prevention of collagen degradation by matrix-metalloproteinases^9^. These effects may be beneficial for a wide variety of resin composite applications in restorative and minimally invasive dentistry, as well as in orthodontics.

A series of studies on experimental composites with systematically varying amounts of BG 45S5 between 0–40 wt% has shown that unsilanized BG fillers can diminish the degree of conversion (DC)^10–12^. Additionally, for the same experimental compositions, BG fillers were demonstrated to impair mechanical properties^11,13^ and increase water sorption and solubility^14^. These properties were affected by the presence of BG fillers through two main mechanisms: (I) a direct effect of unsilanized, hydrophilic and water-soluble BG particles, which cannot reinforce the material structure, and (II) an indirect effect of BG fillers mediated by the DC reduction. The former effect is inherent to BG 45S5 fillers, which are highly soluble and must not be silanized in order to maintain their ion-releasing capability^15^. On the other hand, the issue of a diminished DC depends on particular material composition and can be resolved by adjusting various compositional variables in order to ensure optimal mechanical properties and biocompatibility^16^.

The maximum attainable DC of resin composites is determined by resin viscosity during polymerization, which is in turn affected by resin composition and interactions between the resin and filler particles^17^. These interactions occur in the resin layer adjacent to the surface of filler particles and are determined by the filler surface area and the type of surface treatment^18^. A higher particle surface area is generally related to lower resin mobility and consequently lower DC^19^. Additionally, unsilanized filler particles that are incapable of chemical linking with the polymeric network tend to improve resin mobility and enhance final DC values^20^. These considerations are inconsistent with previous reports of DC being diminished by replacing smaller silanized inert fillers with larger unsilanized BG particles^10–12^ unless the polymerization was affected by some phenomenon other than mobility restrictions. To explain these findings, the capability of unsilanized fillers for the inhibition of polymerization through inactivation of free radicals should be considered^21,22^. The polymerization-inhibiting potential of glass fillers is usually not observed in conventional composites because their fillers are coated with a silane layer that successfully prevents the inhibitory effect^23^. However, the free radical-mediated polymerization of various resins has been shown to be inhibited by unsilanized glass fillers, which consequently leads to an inferior DC^24,25^.

Under clinical conditions, resin composites are light-cured at approximately room temperature and subsequently post-cured at body temperature^26^. By affecting the mobility of reactive species in the polymerizing resin, these temperature levels determine the final extent of polymerization^27^, with DC values ranging between 50 and 90%^28^. Exposing the composite to higher post-cure temperatures improves the mobility of reactive species and increases the final DC^29^. Therefore, post-cure heating above the glass transition temperature of methacrylate polymers can be used to diminish mobility restrictions^30^ and evaluate the hypothesized direct effect of unsilanized BG fillers on DC. As the direct inhibitory effect of BG is not likely to be mitigated by the temperature increase, post-cure heating can be used to make it distinguishable from the temperature-dependent effect of mobility limitations. To the best of our knowledge, such an approach has not been reported up to date.

To separate the direct effect of BG fillers from the effect of restricted mobility, this study investigated DC values of experimental composites that were attained by post-curing at either 37 °C or 150 °C. Resin composites containing 0, 20, or 40 wt% of BG were prepared using three different resin systems and the following null hypotheses were tested: (I) the DC would not be affected by the addition of BG fillers, (II) the effect of BG on the DC would not differ among resin systems, and (III) the DC would not be affected by post-cure heating at 150 °C.

## Materials and Methods

### Experimental resin composites

Experimental composites containing 0, 20, or 40 wt% of BG were prepared with base resins Bis-GMA, Bis-EMA, and UDMA (Table 1). Relative ratios of base and diluent monomers were adjusted in order to allow incorporation of 70 wt% of fillers. The prepared resin systems had similar compositions as those used in commercial resin composites^31^. The resin systems were rendered photoactive by adding 0.2 wt% of camphorquinone (Merck, Darmstadt, Germany) and 0.8 wt% of ethyl-4- (dimethylamino) benzoate (Merck) followed by mixing using a magnetic stirrer for 48 h. The obtained photoactive resin systems were mixed with fillers using a dual asymmetric centrifugal mixing system (Speed Mixer TM DAC 150 FVZ, Hauschild & Co. KG, Hamm, Germany) at 2700 rpm. To avoid overheating of composite pastes, the mixing was performed in five one-minute intervals that were separated by one-minute breaks. After mixing, the prepared composites were deaerated in vacuum during 12 h.

**Table 1:** Composition of experimental resin composites.

Base resin	Material designation	Filler composition (wt%)		Total filler ratio (wt%)	Resin		Filler load (vol%)
		Bioactive glass	Reinforcing fillers (Ba:Si = 2:1)		wt%	Composition (wt%)	
Bis-EMA	BG-0	0	70	70	30	60% Bis-EMA 40% TEGDMA	47
	BG-20	20	50	70	30		50
	BG-40	40	30	70	30		51
Bis-GMA	BG-0	0	70	70	30	60% Bis-GMA 40% TEGDMA	48
	BG-20	20	50	70	30		51
	BG-40	40	30	70	30		52
UDMA	BG-0	0	70	70	30	80% UDMA 20% TEGDMA	47
	BG-20	20	50	70	30		50
	BG-40	40	30	70	30		51

Bioactive glass 45S5: SiO_2_ 45 wt%, Na_2_O 24.5 wt%, CaO 24.5 wt%, P_2_O_5_ 6 wt%, particle size (d50/d99 [µm]): 4.0/13.0, silanization: none, product name/manufacturer: G018-144/Schott, Mainz, Germany.

Barium-fillers (Ba): SiO_2_ 55.0 wt%, BaO 25.0 wt%, B_2_O_3_ 10.0 wt%, Al_2_O_3_ 10.0 wt%, particle size (d50/d99 [µm]): 1.0/4.0, silanization 3.2 wt%, product name/manufacturer: GM27884/Schott.

Silica-fillers (Si): SiO_2_ ≥ 99.8 wt%, primary particle size: 12 nm, silanization 4–6 wt%, product name/manufacturer: Aerosil DT/Evonik Degussa, Germany.

Bis-EMA: ethoxylated bisphenol A dimethacrylate, Esstech, PA, USA; Bis-GMA: bisphenol A glycidyl methacrylate, Esstech; TEGDMA: tri-ethylene glycol dimethacrylate, Esstech; UDMA: urethane dimethacrylate, Esstech.

### Composite specimen preparation

**Figure 1:**
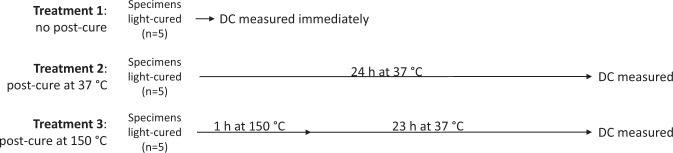
Experimental design.

Composite specimens for the DC evaluation were prepared in cylindrical Teflon moulds (diameter: 6 mm, height: 2 mm). The moulds were filled with the composite, covered from both sides with a polyethylene terephthalate (PET) film and photoactivated for 20 s using a light-emitting diode (LED) curing unit (Bluephase G2, Ivoclar Vivadent, Schaan, Liechtenstein; wavelength range of 380–515 nm, radiant exitance of 1185 mW/cm^2^, as measured using integrating sphere, IS, Gigahertz-Optik GmbH, Puchheim, Germany and spectrometer HR4000, Ocean Optics, Dunedin, FL, USA). The curing unit tip was positioned perpendicularly above the upper mould aperture, immediately adjacent to the composite surface, resulting in a total radiant exposure received by the specimen surface of 23.7 J/cm^2^. Light-curing was performed at room temperature (22 ± 1 °C) and specimen handling was done in a dark environment. Three groups of specimens were prepared according to different post-cure treatments, as shown in Fig. 1. Post-cure heating at 37 ± 1 °C was performed using an incubator (Cultura, Ivoclar Vivadent), whereas post-cure heating at 150 ± 5 °C was performed in a laboratory oven. Both post-cure heating treatments were performed under dry conditions.

After each type of treatment, the DC was measured at the top (0 mm) and bottom (2 mm) surfaces of unpolished cylindrical specimens. Five specimens per experimental group were prepared. For Treatment 1, two separate groups of specimens were prepared for the measurements at 0 mm and 2 mm in order to avoid subsequent DC measurements on freshly prepared specimens with ongoing post-cure polymerization^32^. For Treatments 2 and 3, the post-cure polymerization was considered to be finished at the time of DC evaluation, thus the same specimens were used for the measurements at 0 mm and 2 mm.

### Raman spectroscopy

Raman spectroscopy was performed using an FT-Raman spectrometer (Spectrum GX, PerkinElmer, Waltham, MA, USA) with an excitation NdYaG laser of 1064 nm wavelength, laser power of 500 mW, and spectral resolution of 4 cm^−1^. The sampling area was a circular spot of 0.4 mm in diameter located at the centre of the selected specimen surface (top or bottom). For each spectrum, 60 scans were recorded with a total collection time of 12 min. Raman spectra of uncured composites (n = 5 per material) were collected using the same parameters. Raman spectra were processed using the Kinetics add-on for Matlab (version 7.5.0, MathWorks, Natick, MA, USA).

The DC was calculated using the relative change in the peak height of the spectral band at 1640 cm^−1^ (aliphatic C = C stretching). The band at 1610 cm^−1^ (aromatic C = C stretching) was used as a reference for the phenyl-ring containing resin systems (Bis-EMA and Bis-GMA), whereas the band at 1458 cm^−1^ (C-H stretching) was used as a reference for the UDMA resin system. DC was calculated according to the following equation:

$$DC( \% )=(1-\frac{{(1640c{m}^{-1}/reference)}_{peakheightaftercuring}\,}{{(1640c{m}^{-1}/reference)}_{peakheightbeforecuring}})\times 100$$

The DC evaluation after post-cure heating bears a risk of volatilizing a certain amount of unreacted monomer molecules^33^, which could artificially increase the DC measured by Raman spectroscopy. To address this issue, a preliminary study was performed in which the mass loss was evaluated using an analytical balance in cured specimens of experimental composites (m = 1 g) after 1 h of heating at 150 °C. The average mass losses (%) of 0.04, 0.07, and 0.06 measured for the Bis-EMA, Bis-GMA, and UDMA resin system, respectively, were considered negligible for the DC measurements performed in the present study.

### Statistical analysis

Considering all factor combinations (9 experimental composites x 3 types of post-cure treatment x 2 layer thicknesses x 5 experimental runs), the total number of measured DC values was 270. Normality of distribution and uniformity of variances were evaluated using Shapiro-Wilk’s and Levene’s tests, respectively. Four-way ANOVA was used to explore the effects of the factors “resin system”, “BG amount”, “post-cure treatment”, and “layer thickness”, as well as their interactions. Within each resin system and post-cure treatment, one-way ANOVA was performed and partial eta-squared values were calculated as a measure of the effect size of varying BG amounts on the DC values. Multiple comparisons among different BG amounts and post-cure treatments were performed using one-way ANOVA with Tukey’s adjustment. The overall level of significance was 5% (p < 0.05). The statistical analysis was performed in SPSS 20 (IBM, Armonk, NY, USA).

## Results

Four-way ANOVA identified a highly significant effect for all factors and their interactions (n = 270, p < 0.001). Of most practical importance was the interaction between the factors “resin system” and “BG amount”, which indicates that the investigated resin systems responded differently to the replacement of inert fillers with BG. Therefore, partial eta-squared values representing the practical significance of the factor “BG amount” were calculated for each combination of factors “resin system” and “post-cure treatment” (Table 2). This analysis was performed using the surface DC values (n = 135), which were considered to represent maximum values attainable for a particular experimental composition. Within each type of post-cure treatment, the practical significance of the BG amount declined in the following order of resin systems: Bis-EMA > Bis-GMA > UDMA. In the UDMA resin system, the influence of the BG amount was statistically significant (p < 0.001) only for DC values measured immediately after curing.

**Table 2:** Partial eta-squared values (p-values in parentheses) representing the practical significance (effect size) of variations in the BG amount as a function of resin system and post-cure treatment.

post-cure treatment	resin system		
	Bis-EMA	Bis-GMA	UDMA
no post-cure	0.936 (<0.001)	0.920 (<0.001)	0.829 (<0.001)
post-cured at 37 °C	0.990 (<0.001)	0.969 (<0.001)	N/A (0.05)
post-cured at 150 °C	0.989 (<0.001)	0.957 (<0.001)	N/A (0.62)

**Figure 2:**
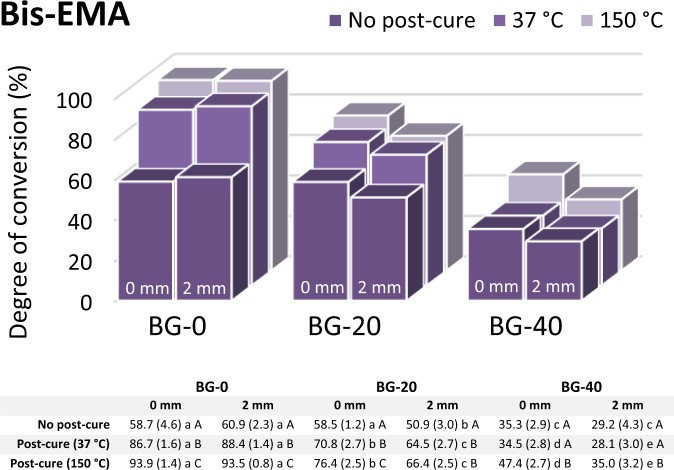
Degree of conversion values (%) for the Bis-EMA resin system (n = 5 per experimental group). Data table below the plot indicates mean values with standard deviations in parentheses. Same lowercase letters denote statistically homogeneous values within rows. Same uppercase letters denote statistically homogeneous values within columns.

**Figure 3:**
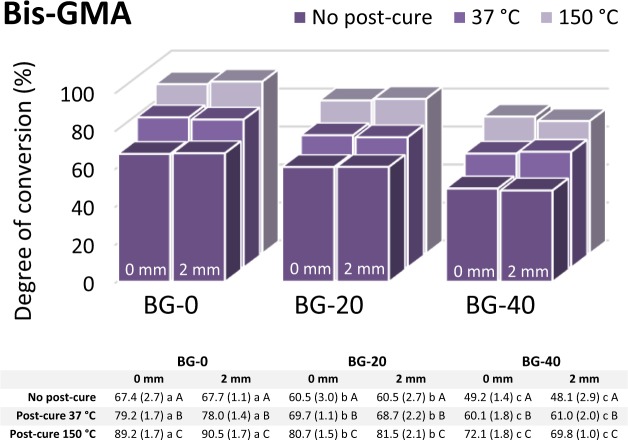
Degree of conversion values (%) for the Bis-GMA resin system (n = 5 per experimental group). Data table below the plot indicates mean values with standard deviations in parentheses. Same lowercase letters denote statistically homogeneous values within rows. Same uppercase letters denote statistically homogeneous values within columns.

**Figure 4:**
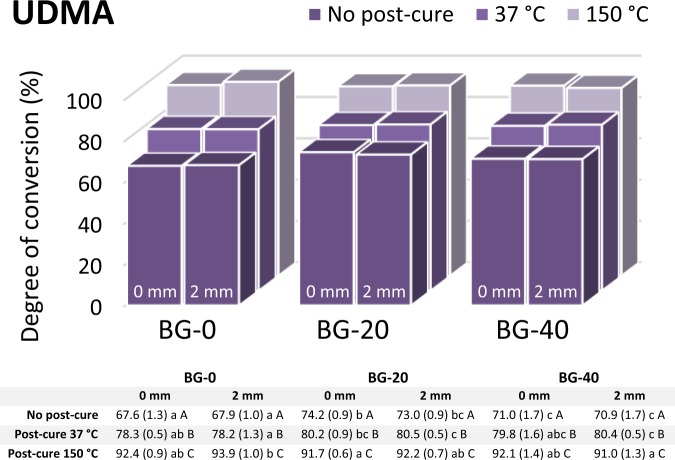
Degree of conversion values (%) for the UDMA resin system (n = 5 per experimental group). Data table below the plot indicates mean values with standard deviations in parentheses. Same lowercase letters denote statistically homogeneous values within rows. Same uppercase letters denote statistically homogeneous values within columns.

The markedly different responses of the three resin systems to the addition of BG can be observed from the DC data presented individually for each experimental group in Figs. 2–4. The strongest effect of the BG amount in the Bis-EMA resin system is represented by a steep DC decline caused by increasing BG amounts (Fig. 2), while a comparatively less pronounced DC decline was identified in the Bis-GMA resin system (Fig. 3). The DC in the UDMA resin system remained unimpaired by the increasing BG amounts (Fig. 4). In fact, the DC measured in the UDMA resin system immediately after curing was even improved by the addition of BG.

In the Bis-GMA and UDMA resin systems, the layer thickness had no effect on the DC, whereas a statistically significant DC decline at 2 mm was identified in the Bis-EMA resin system containing 20 and 40 wt% of BG (p < 0.001 and p = 0.002, respectively).

The post-cure DC increase was observed for all combinations of the resin system and BG amount, except for the specimens of the Bis-EMA resin system with 40 wt% of BG that were post-cured at 37 °C. The DC values attained by post-curing at 150 °C were significantly higher (p < 0.001) than those reached at 37 °C for all experimental composites. In general, the magnitude of the DC improvement due to post-curing at 150 °C (compared to 37 °C) increased in the following order of resin systems: Bis-EMA < Bis-GMA < UDMA (Figs. 2–4).

## Discussion

This study is a sequel to previous studies, which indicated that the addition of BG can diminish the DC of experimental resin composites^10–12^. Post-cure heating at 150 °C was used to distinguish a direct (temperature-independent) effect of BG from an indirect (temperature-dependent) effect of restricted mobility of reactive species. As the DC was reduced by the addition of BG fillers and the reduction varied among resin systems, the first two null hypotheses were rejected. Also, a material-dependent DC improvement was attained by post-cure heating at 150 °C, leading to the rejection of the third null hypothesis.

The DC of resin composites is determined by an interplay of multiple factors affecting the mobility of reactive species throughout polymerization. These factors include viscosity and reactivity of the resin system^31^, interfacial surface area between filler particles and the resin^19^, and temperature at which polymerization occurs^27^. In the case of the experimental composites used in this study, these considerations are additionally complicated by the tendency of nanometre-sized silica particles to agglomerate into larger secondary particles^34^, which is difficult to control experimentally, as well as the fact that resin/filler interactions vary according to different levels of filler silanization^35^. Theoretical predictions about the effect of various filler features on polymerization are furthermore perplexed by the free radical-inactivating potential of unsilanized fillers^17^. To evaluate the integrated effect of multiple factors on the DC of experimental composites filled with BG, the present study employed a full-factorial model with the following variables: resin system, BG amount, layer thickness, and post-cure treatment.

Within each resin system, the materials containing only reinforcing fillers (denoted as BG-0) had the highest filler surface area. The fact that these materials reached DC values of 78–88% after 24 h at 37 °C indicates that all resin systems were sufficiently mobile to reach the extent of polymerization comparable to high-end values reported for commercial composites^28^. Replacing comparatively smaller inert fillers with larger BG particles decreased the total filler surface area; however, the enhanced DC values due to improved resin mobility were identified only for the UDMA resin system. In contrast, the DC for the Bis-EMA and Bis-GMA resin systems was significantly diminished by the replacement of inert fillers with BG. Partial eta-squared values in Table 2 suggest that DC was most strongly affected by BG in the Bis-EMA resin system, followed by the Bis-GMA resin system. On the other hand, the statistically significant effect of the BG amount and the corresponding partial eta-squared values for the UDMA resin system reflect the aforementioned DC improvement due to the decrease in filler surface area.

The potential of unsilanized fillers for the inhibition of polymerization mediated by free radicals has been known for at least four decades^23,25^. Several studies published by different research groups have implied that unsilanized BG fillers can impair the DC of methacrylate resins^10,36,37^. Also, a series of studies performed on the experimental BG-containing composites similar to those based on the Bis-GMA resin system in the present study has indicated that unsilanized BG fillers can diminish the DC in a dose-dependent manner^10–12^. To investigate this issue further, this study employed post-cure heating at 150 °C. Namely, the polymerization that has stopped at room temperature due to the viscosity increase can be restarted by post-cure heating, because unreacted C = C bonds and free radicals on chain-ends are still available in the immobilized reaction medium^29^. Exposing the cured composite to temperatures above the glass transition temperature of the polymer is expected to improve the DC^38^, unless the newly mobilized radicals are being inactivated by unsilanized filler particles. As the hypothesized free radical inactivation competes with the continuation of polymerization, the relative strengths of these two processes should determine the extent of DC improvement attained by post-cure heating^21^. If the free radical inactivation dominates, the capability of post-cure heating for improving DC will be limited. This is a plausible explanation for a limited potential of post-cure heating for improving the DC values of BG-containing composites based on the Bis-EMA resin system (Fig. 2). For the BG-containing composites based on the Bis-GMA resin system, the corresponding DC improvement was slightly more extensive (Fig. 3); however, it was still insufficient for reaching the DC values of the control material (BG-0). Therefore, the DC for the Bis-GMA and Bis-EMA resin systems appear to be limited by a direct effect of BG rather than by immobilization of reactive species.

In contrast to the findings for the Bis-EMA and Bis-GMA resin systems, the DC values in the UDMA resin system were not diminished by the addition of BG (Fig. 4). Unlike Bis-EMA and Bis-GMA, UDMA has a distinct capability to polymerize through chain transfer reactions. By this reaction mechanism, the growth centre at the end of a polymeric chain can be transferred to -NH- groups of the UDMA molecule, rendering that molecule a new initiation site. In that way, chain growth sites can migrate onto new monomer molecules instead of being immobilized on long and crosslinked polymeric chains^39^. Therefore, chain transfer reactions improve the reactivity of UDMA and allow it to reach higher final DC than would be expected considering its glass transition temperature^40^. The improved reactivity of UDMA also causes an enhanced polymerization rate in the early phase of polymerization and the formation of a more densely crosslinked network than would be possible if the polymerization occurred only through C = C groups^16^. It can be speculated that this alternative pathway of polymerizing through -NH- groups can render the UDMA resin system less susceptible to polymerization inhibition by unsilanized BG fillers.

The extent of post-cure polymerization generally depends on the initial DC attained during light-curing, whereby lower initial DC values are accompanied by a higher post-cure DC increase^41^. The post-cure continuation of polymerization mainly depends on resin mobility and the availability of active chain growth centres^42^. The fact that the experimental composites with the lowest initial DC values (BG-20 and BG-40 based on the Bis-EMA resin) showed only a modest post-cure DC improvement, even when resin mobility was improved by heating at 150 °C, adds to the evidence that polymerization was indeed limited by the inactivation of free radicals. Although less pronounced, this phenomenon was also observed for the Bis-GMA resin system, for which no “compensatory” increase in the post-cure polymerization was identified as a response to a lower initial DC due to the addition of BG fillers.

The DC measured 24 h post-cure at 37 °C is commonly regarded as the final value that composite materials reach in clinical application^43^. In the present study, the effect of BG on these DC values varied considerably among resin systems. The Bis-EMA resin system was most affected, for which the maximum BG amount (40 wt%) led to a clinically unacceptable DC of 35%. Comparatively better DC values for the maximum BG amount were measured in the Bis-GMA resin system (~60%) and the UDMA resin system (~80%). After heating at 150 °C, the composites with the maximum BG amount reached DC values of 47%, 72%, and 92% for Bis-EMA, Bis-GMA, and UDMA resin system, respectively. From a practical standpoint, these data imply that the resin systems which were more affected by BG inhibition were also less capable of achieving DC improvement through post-cure heating.

The measuring depths of 0 and 2 mm were selected in order to evaluate curing effectiveness at a clinically realistic layer thickness. For all composites based on the Bis-GMA and UDMA resin system, the DC values measured at 0 and 2 mm were statistically similar, indicating that the radiant exposure of 23.7 J/cm^2^ was sufficient for a homogeneous cure throughout a 2-mm layer. These results are in contrast with a report of a heterogeneous cure for similar experimental composites based on the Bis-GMA resin system, which were cured with a comparable radiant exposure^10^. The discrepancy in findings can be explained by the fact that in the present study comparatively larger specimens were cured in moulds of lower thermal conductivity (Teflon instead of aluminium), leading to a higher temperature rise and consequently higher DC^40^. For the Bis-EMA resin system, the significantly lower DC at 2 mm compared to that at 0 mm can be attributed to a lower number of initiation sites available at the layer bottom due to the combined effect of free radical inhibition by BG fillers and a decline in light transmittance due to the filler/resin refractive index mismatch. A comprehensive report on the latter effect will be published in a subsequent article. It should also be noted that the filler/resin refractive index mismatch cannot fully explain the DC reduction in the Bis-EMA and Bis-GMA resin systems which was identified on the specimen top surface (0 mm) where light attenuation due to scattering was negligible.

Considering the phenomenon of polymerization inhibition reported in this study, as well as in previous studies^10–12^, it should be noted that there are also reports of various types of BG being successfully incorporated into methacrylate resins with no negative effect on the DC^3,36,44^. These studies have investigated various experimental materials, including Bis-GMA/TEGDMA resin filled with bismuth-oxide-modified BG^3^, Bis-EMA/TEGDMA resin filled with zinc-polycarboxylated BG and BG 45S5^36^, and commercial adhesive systems doped with niobium phosphate-modified BG^44^. The fact that the effect of BG on polymerization apparently varies with the compositions of BG, inert fillers, and resin systems indicates the need for further investigations.

In this *in vitro* study, post-cure heating at 150 °C for 1 h was performed to maximize the effect of reactive species mobilization without compromising the accuracy of DC measurements^30^. From a clinical standpoint, post-cure heating at 150 °C is not applicable for improving the DC of composite materials used for direct restorations or orthodontic bonding. However, it is plausible that post-cure heating of indirect restorations based on BG-containing composites would be beneficial for improving their mechanical properties and biocompatibility^29,30^.

The main limitation of the present study is the fact that the inhibitory effect of BG on polymerization was not further characterized by methods based on quantifying free radicals^21^. This should be done in further studies in order to confirm the hypothesized mechanism of free radical inactivation. Also, evaluating only six experimental BG-containing composites precludes generalizations regarding other possible material formulations. However, the basic components of materials investigated in this study are representative of those commonly used in experimental and commercial remineralizing resin composites^3–9,45^. This fact makes the findings of the present study relevant for further compositional adjustments of BG-containing resin composites.

## Conclusions

The potential of bioactive glass 45S5 to adversely affect the degree of conversion of experimental composites was highly dependent on the resin system. The highest reduction in the degree of conversion was observed in the Bis-EMA resin system, which was followed by the Bis-GMA resin system. In contrast, the degree of conversion for the UDMA resin system was not compromised by the addition of bioactive glass. Improving the mobility of reactive species by post-cure heating at 150 °C showed limited potential for increasing the degree of conversion in the Bis-EMA and Bis-GMA resin systems, indicating a direct inhibitory effect of bioactive glass on polymerization.

## Data Availability

The datasets generated during and/or analysed during the current study are available from the corresponding author on reasonable request.
